# Hyperinsulinaemia and hyperglycaemia promote glucose utilization and storage during low- and high-intensity exercise

**DOI:** 10.1007/s00421-019-04257-9

**Published:** 2019-11-09

**Authors:** Hamid Mohebbi, Iain T. Campbell, Marie A. Keegan, James J. Malone, Andrew T. Hulton, Don P. M. MacLaren

**Affiliations:** 1 Prof (Emeritus), Research Institute for Sport and Exercise Sciences, Liverpool John Moores University, Tom Reilly Building, Byrom Street Campus, Liverpool, L3 2AF UK; 2 Sport Science and Faculty of Physical Education, University of Guilan, Rasht, Iran; 3 Department of Anaesthesia, Wythenshawe Hospital, Manchester, UK; 4 School of Health Sciences, Liverpool Hope University, Liverpool, UK; 5 School of Biosciences and Medicine, University of Surrey, Guildford, UK

**Keywords:** Glucose, Insulin, Severity of exercise, Glucose utilization, Carbohydrate oxidation, Non-oxidative glucose disposal, Muscle glycogen

## Abstract

**Purpose:**

The effect of hyperglycaemia with and without additional insulin was explored at a low and high intensity of exercise (40% vs 70% *V*O_2peak_) on glucose utilization (GUR), carbohydrate oxidation, non-oxidative glucose disposal (NOGD), and muscle glycogen.

**Methods:**

Eight healthy trained males were exercised for 120 min in four trials, twice at 40% *V*O_2peak_ and twice at 70% *V*O_2peak,_ while glucose was infused intravenously (40%G; 70%G) at rates to “clamp” blood glucose at 10 mM. On one occasion at each exercise intensity, insulin was also infused at 40 mU/m^2^/per min (i.e. 40%GI and 70%GI). The glucose and insulin infusion began 30 min prior to exercise and throughout exercise. A muscle biopsy was taken at the end of exercise for glycogen analysis.

**Results:**

Hyperglycaemia significantly elevated plasma insulin concentration (*p* < 0.001), although no difference was observed between the exercise intensities. Insulin infusion during both mild and severe exercise resulted in increased insulin concentrations (*p* < 0.01) and GUR (*p* < 0.01) compared with glucose (40%GI by 25.2%; 70%GI by 26.2%), but failed to significantly affect carbohydrate, fat and protein oxidation. NOGD was significantly higher for GI trials at both intensities (*p* < 0.05) with storage occurring during both lower intensities (62.7 ± 19.6 g 40%GI; 127 ± 20.7 g 40%GI) and 70%GI (29.0 ± 20.0 g). Muscle glycogen concentrations were significantly depleted from rest (*p* < 0.01) after all four trials.

**Conclusion:**

Hyperinsulinaemia in the presence of hyperglycaemia during both low- and high-intensity exercise promotes GUR and NOGD, but does not significantly affect substrate oxidation.

## Introduction

Carbohydrates and fats are the two major energy sources that fuel muscle during prolonged exercise. The fatigue associated with prolonged performance has been reported to coincide with the depletion of endogenous stores of carbohydrate, and of disturbances in the level of circulating plasma glucose (Cermak and van Loon 2013). Significant improvements in endurance performance and capacity are well established when carbohydrates are ingested before and/or during activity (Stellingwerff and Cox 2014). These improvements could be due to a number of factors such as stimulation of carbohydrate receptors in the oral cavity modulating neural drive and attenuating perceived exertion (Carter et al. 2004) and/or maintenance of plasma glucose concentration leading to an increase in carbohydrate oxidation late in exercise (Coggan and Coyle 1987; Jeukendrup 2004). In addition, it has been demonstrated that carbohydrate intake during exercise not only increases oxidation of carbohydrate, but may spare use of muscle glycogen and thereby improve performance or time to fatigue (Stellingwerff et al. 2007; Tsintzas et al. 1995). However, a number of studies have failed to show a sparing effect on muscle glycogen (Coyle et al. 1986; Mitchell et al. 1989).

Stellingwerff and Cox (2014) proposed a likelihood of performance benefits with carbohydrate ingestion when exercise was longer than 2 h, but not necessarily if the bout was less than 1 h. They concluded that the primary mechanism by which carbohydrates enhance endurance performance was due to a high rate of carbohydrate delivery (> 90 g/h) resulting in elevated rates of carbohydrate oxidation. Consequently, many investigations have explored the promotion of carbohydrate delivery to muscle by using high levels of a single source of carbohydrate or by ingesting multiple transportable carbohydrates such as glucose:fructose combinations (Newell et al. 2018). The issue with ingesting large amounts of carbohydrate during performance (particularly running) is that the gastrointestinal system is compromised and leads to unwarranted symptoms such as gut pain, flatulence, diarrhoea, and vomiting. Even so, it appears that the maximum rate of exogenous carbohydrate is achieved when ingesting around 90 g/h. Amounts of ingested carbohydrate at these high levels results in a maximal rate of exogenous carbohydrate oxidation of ~ 1.0 g/min for single sources of carbohydrates or ~ 1.75 g/min using multiple transportable carbohydrates (Jeukendrup 2010).

But an intriguing question remains as to what is the actual maximal rate that exogenous carbohydrate can be oxidized during exercise? Since the gut presents a ‘barrier’ not just in terms of carbohydrate delivery into the blood but also in relation to gastrointestinal problems, any question as to the maximal potential rates of exogenous carbohydrate utilization during exercise are thereby hindered by the gut. However, infusing glucose directly into a vein disposes of the need for gut transport and other inherent problems. Previously, we have employed the hyperglycaemic glucose clamp technique to observe metabolic changes during intense bouts of exercise (MacLaren et al. 1999). In this study, we observed that maintained hyperglycaemia resulted in a maximal glucose utilization rate (GUR) of 1.8 g/min (i.e. 108 g/h) and a maximal rate of total CHO oxidation of 2.65 g/min. Therefore, ~ 70% of the exogenous carbohydrate was oxidized, the rest of the carbohydrate oxidation arising from endogenous sources (most probably muscle glycogen). In fact, two of our younger participants presented with a GUR of ~ 2.8 g/min (168 g/h) which is similar to data previously reported (Coyle et al 1991; Hawley et al 1994). It would thus be reasonable to suggest that the ~ 1 g/min higher rate of exogenous glucose use from infusion compared with ingestion studies is, in part, due to the gut as a ‘barrier’.

Recently, we observed higher rates of GUR and CHO oxidation in young athletes compared with elderly athletes (Malone et al 2019). In part, some of the variation may have been due to a degree of insulin resistance with the elderly participants. It may be possible to further stimulate GUR and CHO oxidation by infusing insulin as well as glucose during exercise.

Insulin levels are normally suppressed during exercise, although they can be increased when ingesting CHO. The combination of increased insulin and exercise is crucial for the enhanced muscle CHO oxidation, since both promote the appearance of GLUT4 on muscle membrane (Kristiansen et al. 1997). However, there is another aspect of insulin that needs to be remembered, and that is the effect of insulin on increasing skeletal muscle blood flow (Baron 1994; Zheng and Liu 2015; Nuutila et al. 2000). Insulin enhances the compliance of arteries, relaxes resistant arterioles to increase tissue blood flow, and dilates capillaries to expand muscle blood volume (Zheng and Liu 2015). In fact, Baron et al (1994) confirmed a coupling of the vascular effects of insulin and its metabolic effects by showing that changes in insulin-mediated leg blood flow were mirrored by changes in insulin-mediated glucose disposal.

The present investigation was undertaken to examine the role of additional insulin as well as maintained hyperglycaemia on carbohydrate metabolism at a low (40% *V*O_2max_) and high (70% *V*O_2max_) exercise intensity. Consequently, we examined the consequences on the total rate of carbohydrate oxidation as measured by respiratory means and the rate of glucose utilization as assessed by the rate of glucose infusion. Furthermore, by determining the difference between the glucose infused and the carbohydrate oxidized it is possible to calculate whether non-oxidative glucose disposal (NOGD) occurred. Additionally, we could observe the effects on muscle glycogen use.

## Methods

### Participants

Eight male athletes provided written informed consent to participate in the study after gaining approval from the South Manchester Research Ethics Committee. The age of the participants was 22.5 ± 7.4 years, height 1.76 ± 0.03 m, weight 72.1 ± 6.0 kg, and *V*O_2peak_ 65.1 ± 10.9 ml kg^−1 ^min^−1^. All completed a pre-participatory exercise health screening questionnaire (Chisholme et al. 1975) and underwent a full medical history and examination. No family history of diabetes was identified, and none of the participants were taking any medication.

### Preliminary testing

Peak oxygen uptake (*V*O_2 peak_) was determined using a progressive incremental exercise test on an electrically braked cycle ergometer (Bosch ERG 551, Robert Bosch GMBH, Berlin, Germany). After a 5-min warm-up at a work load of 90 W, the test commenced at 180 W, and the power output increased by 30 W increments every 2 min until volitional exhaustion. Expired air was analysed using an automated gas analysis system (P.K. Morgan, Chatham UK). The results were used to calculate the power outputs corresponding to 40 and 70% of *V*O_2 peak_. After a suitable rest period, the participants underwent a familiarization exercise where power outputs were adjusted to produce *V*O_2_, as measured, corresponding to 40 and 70% of *V*O_2 peak_. These power outputs were used in the definitive experiments.

### Experimental design

**Fig. 1:**
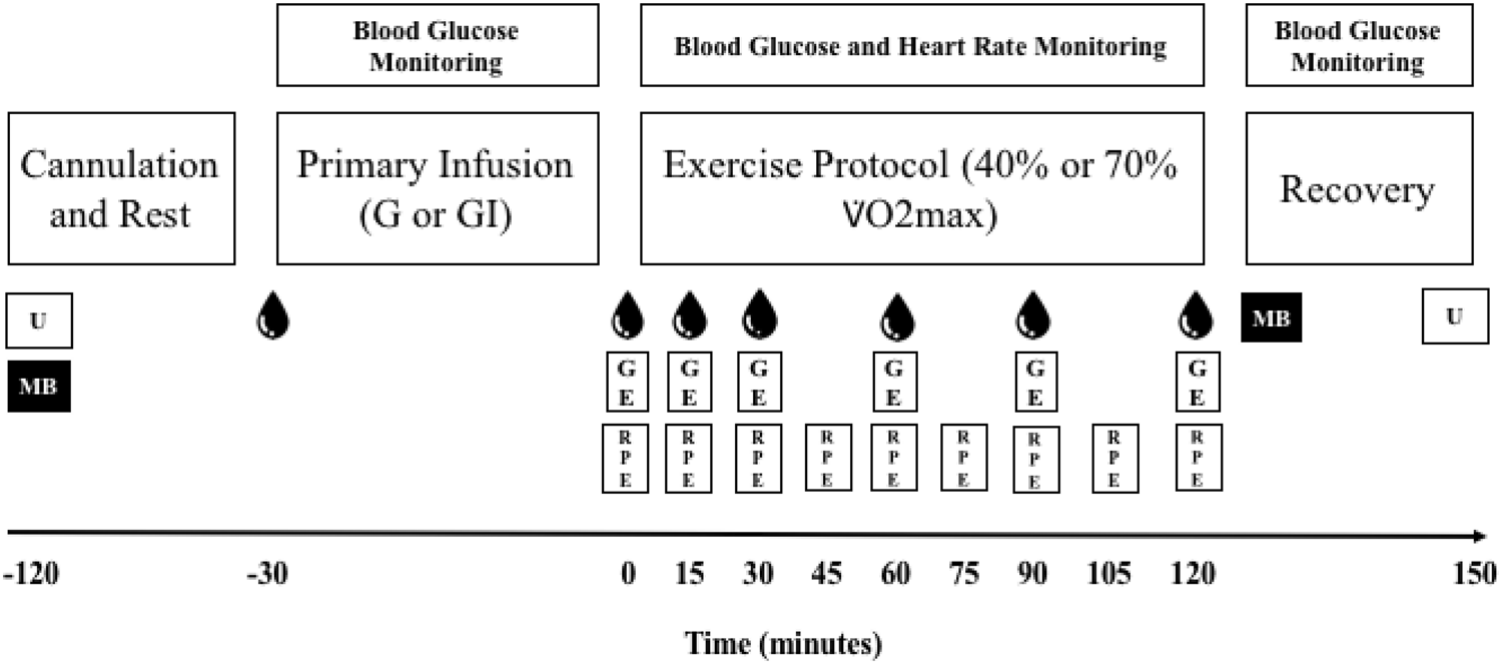
The experimental protocol. After an overnight fast, the participant arrived in the laboratory without breakfast at 08:30 h. Participant voided before starting cannulation

Each participant undertook four trials on separate days, 3–4 weeks apart. They cycled twice at 40% *V*O_2 peak_ and twice at 70% *V*O_2peak_. On all occasions, 20% (w/v) d-glucose (Baxter Healthcare, Thetford, UK) was infused intravenously to raise and maintain blood glucose at 10 mM. On one occasion, at each exercise intensity, insulin was infused concurrently with the glucose. Oxygen consumption and VCO_2_ were measured at rest and at intervals during the 120 min of exercise and blood was taken for measurement of hormone and metabolite concentrations. A muscle biopsy was performed at the end of the exercise, and a control resting biopsy on a separate occasion, for glycogen content. Figure 1 illustrates the design.

### Experimental protocol

Participants reported to the laboratory after an overnight fast at 8.30 am. After voiding the bladder, a 16 g cannula (Venflon, Beckton Dickinson, Oxford, UK) for glucose infusion was placed into a forearm vein under local anaesthetic. A second (20 g) cannula, for blood sampling, was inserted retrogradely into a vein on the other forearm, under local anaesthesia, and maintained patent with a slow (10 ml/h) infusion of 0.9% saline.

After resting for 30 min, a baseline blood sample was taken for analysis of insulin. Oxygen consumption and VCO_2_ were measured. The infusion, glucose (G) or glucose and insulin (GI), was then commenced with the participant remaining at rest for a further 30 min. Oxygen consumption and VCO_2_ were measured and blood taken between 25 and 30 min (time “0” on the figures). The participants then exercised at 40% or 70% *V*O_2peak_ for 120 min. Gas exchange was measured for the first 5 min and blood samples taken, and thereafter gas exchange measured at 15, 30, 60, 90 and 120 min of the exercise period. Heart rate was measured throughout using a PE300 Heart Rate Monitor (Polar Electropolar OY, Kempele, Finland). Rating of perceived exertion (RPE) was recorded at 15-min intervals using the Borg RPE 1–20 scale (Borg 1982). The order of the trials, 40% or 70% with or without insulin, was randomized.

### Glucose and insulin infusions

20% d-glucose was delivered via a volumetric infusion pump (Colleague Infusion Pump, Baxter, Thetford, UK) and blood glucose was raised to 10 mM using the algorithm of DeFronzo et al. (1979). Blood thereafter was taken at 5-min intervals and the infusion rate adjusted empirically to maintain blood glucose at a target of 10 mM for the remainder of the rest period and throughout exercise. Blood glucose analysis was performed using a Haemocue Blood Glucose Analyser (Haemocue AB Ltd, Angelholm, Sweden) based on a photometric evaluation using a modified glucose dehydrogenase method on whole blood. On one occasion at 40% *V*O_2peak_ and on one occasion at 70% *V*O_2peak_, insulin (Humulin S, Eli Lilly Ltd, UK) was delivered via a side arm on the glucose infusion by a syringe driver (Graseby 3100, Smith Medical, Watford, UK). Insulin infusion was started at the same time as the glucose. An initial priming dose was given in accordance with the algorithm of DeFronzo et al (1979), followed by an infusion at 40 mU m^2^ min^−1^.

Immediately post-exercise, a muscle biopsy was taken from the vastus lateralis using the conchotome technique, and on a completely separate occasion 1 week after completion of the trials a resting biopsy was taken (MacLaren et al 1999). Samples were placed in Eppendorf tubes which were frozen in liquid nitrogen, and then immediately stored at − 70 °C until future analysis of glycogen content. The bladder was voided, with the urine volume measured and a sample retained for analysis for glucose and nitrogen. Changes in blood urea pool were calculated from body weight and changes in plasma urea.

No formal assessment was made of dietary intake, but the participants were instructed to maintain the same diet for the 2 days prior to each experimental visit, as well as for the resting biopsy, and to refrain from strenuous exercise, alcohol and caffeine for 24 h prior to each test.

## Blood sampling and analysis

Blood was taken for insulin at baseline, after the 30-min priming infusion and at 15, 30, 60, 90 and 120 min of exercise. Plasma insulin was determined using a double antibody radioimmunoassay kit (Pharmacia and Upjohn, Milton Keynes, UK). Glucagon concentrations were determined using a glucagon RIA kit (Diagnostic Products Corporation, Lllanberis, UK).

Muscle biopsy samples were analysed for glycogen content in tissue homogenated with an enzymatic method after acid hydrolysis of the tissue (Lowry and Passonneau 1972). A bicinchoninic acid method was used for protein determination (Smith et al. 1985).

The urine, collected over the whole experiment, was analyzed for glucose using the hexokinase method (Hexokinase kit, Sigma-Aldrich, UK), and nitrogen content using the Kjeldahl technique.

## Calculations

Exogenous glucose utilization was calculated as the glucose infused, corrected for glucose lost in the urine (DeFronzo et al. 1979), and averaged over 20-min epochs.

$${\text{GUR }} = \, \left( {D/\left( {W \, \times \, 20} \right)} \right) \, {-} \, U \, {-} \, \left( {\left( {g2 \, {-} \, g1} \right) \, \times \, 0.0095} \right) \, \times \, 1000,$$

where *D* is the total glucose delivery (mol/min), *W* the body weight (kg), *U* the urinary glucose loss (mM/kg/min), *g*2 the blood glucose at the end of a 20-min epoch (mM), *g*1 blood glucose at the start of a 20-min epoch (mM) (*g*2 − *g*1) × 0.0095 = space correction factor, 20 = time correction.

Non-oxidative glucose disposal was determined by the difference between glucose utilized and carbohydrate oxidized.

Carbohydrate (CHO) and fat oxidation rates were calculated from the gas exchange data using stoichiometric equations (Frayn 1983). Non-protein respiratory exchange ratio (RER) was calculated by correcting for urinary nitrogen excretion (UNE) and blood urea nitrogen content (BUN) by having participants void at the start of the experiment and again on completion of the 2-h exercise (Frayn and Macdonald 1997). Protein oxidation was calculated from the measurements of N in the urine by the method of Kjeldahl, correcting for changes in body urea pool. The quantity of urinary nitrogen excreted and changes in blood urea were used to calculate the total amount of protein oxidized (Nair 1997); a constant rate of protein oxidation was assumed over the period of urine collection.

### Statistical analysis

Two-way ANOVA for repeated measures was employed to evaluate the differences between trials over time. Statistically significant differences at specific time points were identified using Tukey’s post hoc test. Areas under the curve were measured to determine total glucose utilization and substrate oxidation, and a *t* test applied to determine the significance of any differences. All results are expressed as a mean ± SEM. The level of statistical significance was taken as *p* < 0.05.

## Results

### Exercise performance

Power output, mean heart rate, RPE and percentage of *V*O_2peak_ achieved are shown in Table 1. In all instances the differences between the 40 and the 70% trials were significant (*p* < 0.001).

**Table 1:** Power output and physiological responses during exercise

	40%GI	40%G	70%GI	70%G
Power output (W)	103.5 ± 6.0	103.5 ± 6.1	187.6 ± 8.5*	187.6 ± 8.5*
Heart rate (bpm)	117.1 + 5.8	113.6 ± 5.4	151.2 ± 4.6*	147.6 ± 6.0*
RPE	10.6 ± 0.4	10.7 ± 0.6	14.4 ± 0.3*	14.2 ± 0.6*
% *V*O_2 peak_	42.0 ± 1.0	40.7 ± 1.0	66.1 ± 2.5*	69.1 ± 5.3*

*Significantly different from 40% trials (*p* < 0.01)

### Glucose and insulin concentrations

**Fig. 2:**
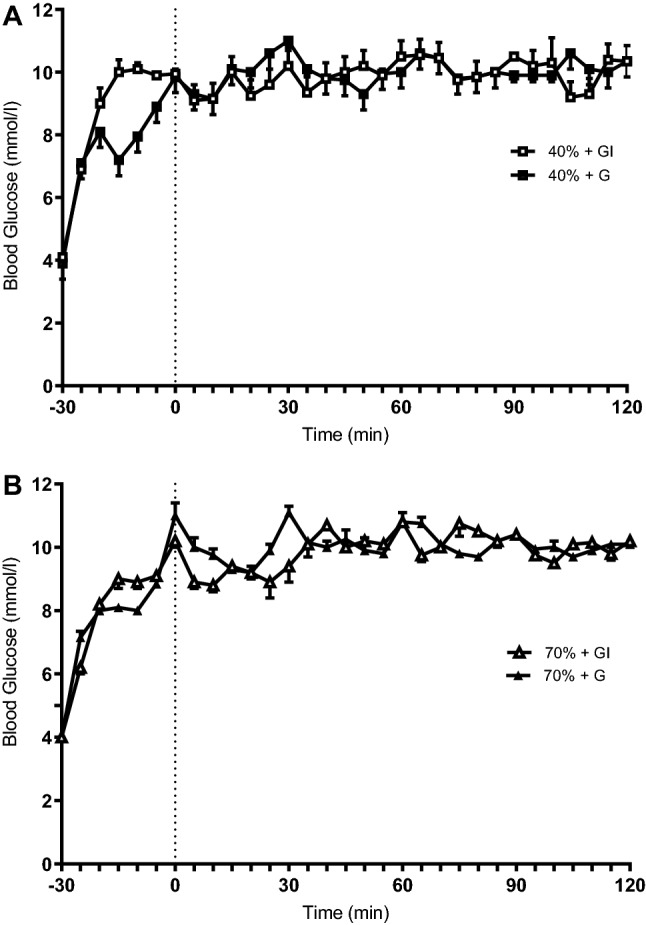
Blood glucose concentrations at rest (− 30), after prime infusion (0), and during 120 min of cycling at 40% *V*O_2_max (**a**) and 70% *V*O_2_max (**b**)

Blood glucose concentrations during the treatments can be observed in Fig. 2. It is clear that the maintained hyperglycaemia of 10 mM was achieved (a CV of 4.6–6.4% was noted for the conditions).

**Fig. 3:**
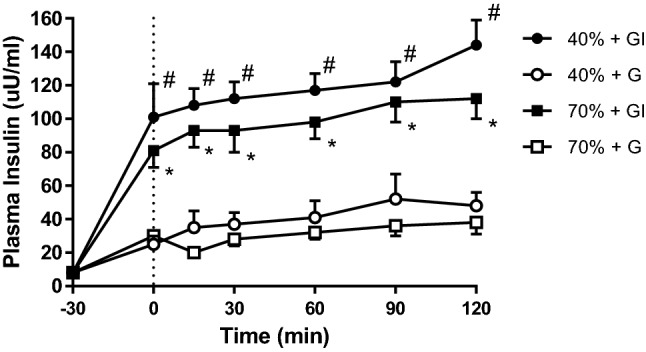
Plasma insulin concentrations at rest (− 30), after prime infusion (0), and during 120 min of cycling at 40% and 70% *V*O_2peak_. ^#^Significant difference between 40%GI and 40%G (*p* < 0.05). *Significant difference between 70%GI and 70%G (*p* < 0.05)

The plasma insulin concentrations achieved are evident in Fig. 3. Insulin concentrations in GI treatments were significantly higher than for G (*p* < 0.001). There were no differences in insulin concentration between the 40% and 70% trials in spite of insulin infusion.

### Glucose utilization rate (GUR)

**Fig. 4:**
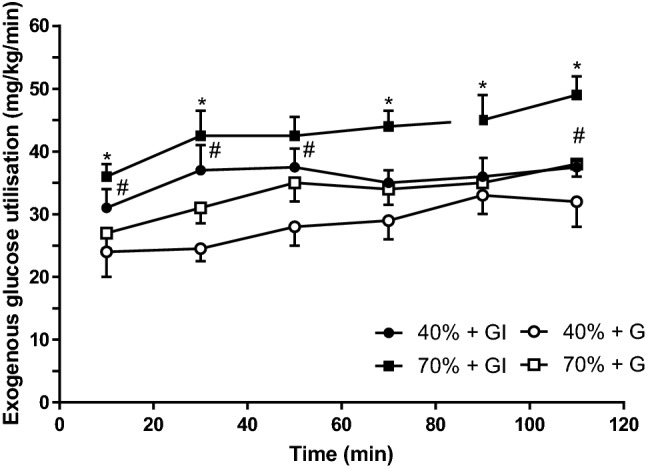
Rates of exogenous glucose utilization during 120 min of cycling at 40% and 70% *V*O_2peak_ under hyperglycaemia (G) and hyperglycaemia + hyperinsulinaemia (GI). * Significant difference from 70%G trials (*p* < 0.05). ^#^Significant difference from 40%G trials (*p* < 0.05)

Figure 4 clearly demonstrates that GUR were significantly higher for 70%GI than for 70%G (*p* < 0.05), and likewise for 40%GI compared with 40%G (*p* < 0.05). The total glucose utilized for each of the trials was 245.0 ± 20.8 g (40%G), 319 ± 23.2 g (40%GI), 288 ± 20.3 g (70%G), and 376 + 22.2 g (70%GI). Significant differences were calculated between the two 40% trials (*p* < 0.05) and the two 70% trials (*p* < 0.01).

### Carbohydrate oxidation

**Fig. 5:**
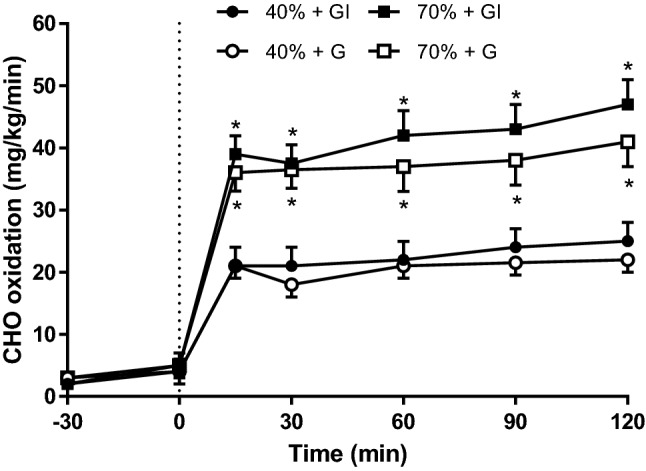
Carbohydrate oxidation at rest (− 30), after prime infusion (0), and during 120 min of cycling at 40% and 70% *V*O_2peak_. *Significant difference from 40% *V*O_2peak_ trials (*p* < 0.05)

The rates of carbohydrate oxidation were significantly higher in both the 70% trials than the 40% trials (Fig. 5), although the addition of insulin made no difference at either exercise intensity. The total carbohydrate oxidized for each trial during the exercise period was 182.0 + 5.0 g (40%G), 192 ± 13 g (40%GI), 324 ± 31.6 g (70%G), and 347 ± 21.7 g (70%GI).

### Non-oxidative glucose disposal (NOGD)

**Fig. 6:**
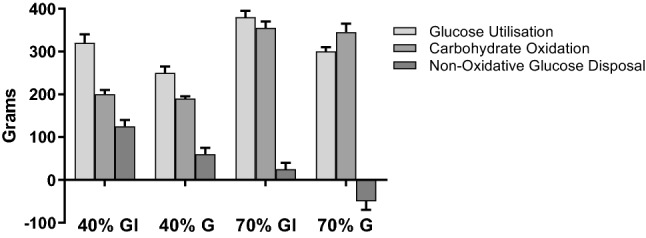
Carbohydrate metabolism (i.e. total glucose utilization, carbohydrate oxidation, and non-oxidative glucose disposal) during 120 min of exercise at 40% and 70% *V*O_2_peak under conditions of hyperglycaemia (G) and hyperglycaemia + hyperinsulinaemia (GI)

Non-oxidative glucose disposal in both the 40% and the 70% trials was significantly increased by insulin (Fig. 6). The values were 62.7 ± 19.6 g (40%G), 127 ± 20.7 g (40%GI), 29.0 ± 20.0 g (70%GI), and − 36.2 ± 30.0 g (70%GI). In 70%G, the carbohydrate oxidized over the 120 min of exercise exceeded the rate of glucose infusion such that non-oxidative disposal was negative i.e. endogenous reserves were utilized, whereas for 70%GI and the two 40% trials, glucose utilization exceeded oxidation and thereby glucose was “stored.”

### Other metabolic changes

Protein oxidation during the 120 min of exercise, derived from urinary nitrogen excretion and changes in blood urea, could only be calculated over the whole time period, and it was expectedly small. Total protein oxidized was 4.55 ± 1.24 g (40%G), 3.37 ± 1.69 g (40%GI), 3.10 ± 1.41 g (70%G), and 3.95 ± 0.63 g (70%GI), respectively, with no difference between trials. These values represent between 0.65 and 1.5% of total energy expenditure during the exercise period.

Total fat oxidation during the 120 min of exercise was 49.20 ± 25.10 g (40%G), 35.0 ± 6.02 g (40%GI), 59.4 ± 9.14 g (70%G), and 41.7 + 8.86 g (70%GI). No significant differences were apparent.

### Muscle glycogen

**Fig. 7:**
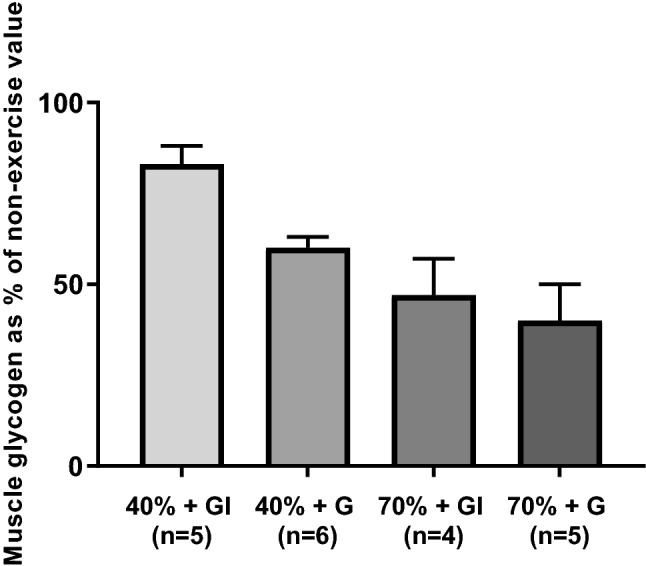
Mean % of muscle glycogen after 120 min of exercise compared with rest values

Unfortunately, it was not possible to persuade all eight participants to have a total of five biopsies, although they all did agree to have at least three biopsies of which one was a resting sample. Results are presented in Fig. 7. The numbers are too small to undertake meaningful statistics, but it can be seen that in both the 40%GI and 70%GI the addition of insulin tended to preserve muscle glycogen.

## Discussion

Hyperglycaemia was maintained throughout the exercise periods with little variation, which emphasizes the suitability of the ‘clamp’ technique and is in keeping with data reported from our previous investigations (MacLaren et al 1999: Malone et al. 2019). This suggests that the GUR reliably assesses the rate of whole body glucose utilization, and that furthermore the differences between GUR and total carbohydrate oxidation reflect glucose disposal.

The combination of hyperglycaemia (10 mM) and hyperinsulinaemia (< 20 U/ml) has been shown to totally suppress liver glucose output throughout 120 min of exercise at 70% *V*O_2peak_ as well as to promote glucose uptake and glucose oxidation (Hawley et al 1994). Furthermore, endogenous glucose production is reduced when insulin is infused, and complete suppression is attained at an infusion rate of 1.0 mU/kg/min or more during mild (~40 *V*O_2max_) exercise (Wasserman et al. 1991). Since net splanchnic uptake is negligible, the brain uptake of glucose is unaffected by insulin or exercise (Ahlborg and Wahren 1972), and as little (1–4%) glucose is taken up by adipose tissue following glucose and insulin administration (Björntorp et al. 1971), the total amount of glucose infused provides a measure of glucose disposal by peripheral tissues (DeFronzo et al. 1979; 1981). Thus, hyperglycaemia (~10 mM) and hyperinsulinaemia (> 20 U/ml) are likely to suppress hepatic glucose production completely, and thereby the rate of glucose infusion needed to maintain blood glucose at ~ 10 mM reflects whole body glucose utilization. The findings from this investigation are supportive of this.

A notable effect of hyperglycaemia was that total carbohydrate oxidation was maintained at a rate in excess of 2.5 g/min during the exercise period at 70% *V*O_2peak_ and 1.4 g/min at 40% *V*O_2peak_. The findings at the higher exercise intensity are similar to those obtained in previous investigations (Coyle et al 1991; MacLaren et al 1999) and appear to be the highest rates observed in exercising humans. It is unlikely that total carbohydrate oxidation rates greater than ~ 3 g/min will be seen. The influence of approximately 60% higher plasma insulin concentration in the GI trials with only a 5% (non-significant) enhancement of carbohydrate oxidation supports the idea that a maximal rate of carbohydrate oxidation was attained.

The effects of hyperglycaemia on fat oxidation are similar to those found in a previous study (MacLaren et al 1999) insofar as a rate of ~ 0.2 g/min was observed. The suppression of fat oxidation is due to elevated insulin, which is a potent anti-lipolytic hormone. The non-significant reduction in fat oxidized at both respective exercise intensities when insulin was infused supports this point. Likewise, the low contribution of protein oxidation to exercise is as expected, since CHO and fat are well recognized as being the major contributors to energy whereas the contribution of protein is small.

Calculations of the contribution of carbohydrate, fat, and protein to the total energy used are remarkably similar for 40%GI, 40%G, and 70%G, whereby hyperglycaemia ensured that carbohydrate oxidation provided ~ 70% of the total energy compared with ~ 28% from fat and ~ 2% from protein. However, 70%GI resulted in 80.8% energy from carbohydrate, 18.7% from fat, and 0.5% from protein. This may seem peculiar since there were no significant differences found between the two 70% trials for carbohydrate or fat oxidation, although examination of the data reveals that the 70%GI trial produced a higher total carbohydrate oxidation than 70%G (347 vs 324 g) and a lower total fat oxidation (41.7 vs 59.4 g). Taken together, these values are reflected in the overall higher total carbohydrate contribution to energy consumption.

In contrast to the similar levels of carbohydrate oxidation observed, GUR was significantly higher during GI than G. Indeed the GUR of 2.4 g/min for 70%G is similar to those obtained in previous studies (Coyle et al 1991; Hawley et al 1994) and for the younger participants in our previous study (MacLaren et al 1999). However, the 3.13 g/min for 70%GI are much higher than previously observed and reflect the highest levels of GUR recorded during exercise.

In the present study, insulin infusion increased GUR both during mild (29.5 ± 2.9 vs. 36.7 ± 2.5 mg/kg/min) and severe exercise (34.3 ± 2.3 vs. 43.5 ± 2.6 mg/kg/min). This represents a remarkably consistent effect in terms of percentage increase in glucose uptake; 24.4% for mild exercise and 26.8% for severe exercise. To put figures for the relative intensities of exercise into context, exogenous GUR values during 70%G and 70%GI exercise were 16.3% and 18.5% higher than during 40%G and 40%GI, respectively. The addition of insulin increased GUR at 40%GI to a rate 6.8% higher than even the 70%G trial!

DeFronzo et al. (1981) reported that 85% of total body glucose metabolism during insulin infusion and exercise can be accounted for by skeletal muscle uptake. The results of this investigation demonstrate that insulin and exercise act synergistically to enhance glucose disposal during both mild and severe exercise in males. Exercise is associated with marked increases in blood flow and capillary surface area to working muscle, which in turn leads to increased uptake of glucose by exercising muscle. DeFronzo et al. (1981) demonstrated that when 30-min mild exercise (40% *V*O_2max_) was combined with hyperinsulinaemia (~75 U/ml), leg blood flow increased approximately ninefold and glucose uptake increased markedly for the same rate of insulin infusion. The interpretation of their findings was that the increase in glucose uptake (for the same insulin level) was mediated by increased blood flow to, and increased capillary surface area in, the exercising muscles. This interpretation was supported by close correlations between the changes in blood flow and glucose uptake, a fact also observed by Baron et al (1994). Exercise and insulin are thus shown to interact synergistically in the control of glucose uptake (DeFronzo et al. 1981; Wasserman et al. 1991).

So it appears that the ~ 25% ‘extra’ carbohydrate utilized under hyperinsulinaemia is probably due to enhanced blood flow and glucose transport to the working muscle. However, not all of the delivered glucose appears to be oxidized, as is reflected in the similar carbohydrate oxidation rates between the GI and G trials. So what happens to the larger amount of glucose? It would appear that storage of the glucose occurs, either as carbohydrate and/or as fat. The findings with respect to NOGD reflect this assertion.

At rest, insulin-mediated glucose disposal occurs by glycogen storage and oxidation in roughly equal proportions (Young et al. 1988), although we have shown that 73% of infused carbohydrate during a hyperglycaemic clamp at rest was stored and 27% oxidized (MacLaren et al 2011). Similar findings have been reported post-exercise (Mikines et al. 1988), and showed that 73.4% of infused glucose was stored and 26.6% oxidized.

The observation of storage occurring during exercise has not been reported previously, since infusion studies have consistently reported a greater rate of total carbohydrate oxidation (Coyle et al 1991; Hawley et al 1994; MacLaren et al 1999; Wasserman et al 1991). However in the present study, NOGD was 38.4%, 24.7%, and 5.6% during 120 min of exercise for 40%GI, 40%G and 70%GI, yet at both exercise intensities exogenous insulin increased GUR by remarkably similar proportions as previously mentioned i.e. 24.4% and 26.2% for 40%GI and 70%GI, respectively. In fact, the rates for NOGD were ~ 1 g/min for 40%GI compared with ~ 0.5 g/min for 40%G and ~ 0.25 g/min for 70%GI. The fact that glucose storage occurred during an exercise intensity of 70% *V*O_2peak_ is quite remarkable and not been reported previously. But does this mean that all the storage is in the form of muscle glycogen?

Muscle glycogen concentrations for 70%G were 41.0% of resting values which implies that 59% underwent glycogenolysis. These rates are comparable with the 55.6% observed by MacLaren et al (1999) and the 56.8% observed by Coyle et al. (1991). On the other hand, muscle glycogen for 40%G, 40%GI, and 70%GI were substantially reduced, i.e. 8%, 20%, and 48% of resting values, respectively. The data for exercise at 40% *V*O_2peak_ reflect the limited use of muscle glycogen at such low intensities but do show that, in spite of NOGD, the glycogen stores are being emptied somewhat. The results from 70%GI are not too dissimilar to that found by Bourey et al (1990), who observed a ~ 50% decrease in muscle glycogen after exercise at 78% *V*O_2peak_ for 60 min, although they employed a euglycaemic hyperinsulinaemic clamp.

It would appear that the mismatch between NOGD and likely muscle glycogen levels points to storage occurring. It is unlikely that the fate of the glucose is towards glycogen synthesis at the higher exercise intensity, since it seems unfeasible that both glycogen storage and breakdown are stimulated simultaneously. It seems more reasonable to suggest that the fate of glucose is towards lipid storage or possibly liver glycogen synthesis. Since we did not undertake analyses of liver glycogen stores or intramuscular or adipose tissue triglycerides, we were unable to determine the fate of NOGD. However, due to the probability that splanchnic blood flow is diminished during exercise and blood flow to exercising muscle is enhanced, it would seem more reasonable to propose that the bulk of the NOGD was for the purpose of promoting muscle lipid stores. Future investigations may be able to furnish the answer.

In this study, “resting” muscle biopsies were taken on an occasion completely separate from the exercise bouts. This may be viewed as an issue since, ideally, the biopsy should have been undertaken at the start and end of each trial. We found this impractical (a) due to the concern of our participants, and (b) the likely effect on subsequent exercise performance due to the biopsy. As it was, care was taken to ensure the participants followed the same dietary and exercise regimen before each of the studies, and this seems to have been successful in producing “resting” muscle glycogen with a relatively small degree of variation between the participants. We have undertaken a similar strategy previously (MacLaren et al 1999).

## Conclusion

This study showed that hyperglycaemia and hyperglycaemia with hyperinsulinaemia resulted in very high rates of carbohydrate oxidation, although there were no apparent differences due to hyperinsulinaemia. Furthermore, the effects of hyperglycaemia and hyperinsulinaemia produced the highest rates of GUR reported during exercise. The resultant elevated GUR from hyperglycaemia, and hyperglycaemia with hyperinsulinaemia caused an elevation of NOGD, especially at the low exercise intensities. The fact that NOGD was observed at an exercise intensity of 70% *V*O_2peak_ when the plasma insulin concentration was very high is quite remarkable and has not been reported previously. The findings in relation to hyperinsulinaemia clearly demonstrate the role of insulin as a ‘storage’ hormone, even during severe exercise. The fact that hyperglycaemia with hyperinsulinaemia appears to attenuate muscle glycogen utilization during low(40% *V*O_2peak_) and probably also during high (70% *V*O_2peak_) exercise intensities would support the view that carbohydrate ‘feeding’ during exercise spares muscle glycogen.
